# BMP-2 and TGF-β Stimulate Expression of β1,3-Glucuronosyl Transferase 1 (GlcAT-1) in Nucleus Pulposus Cells Through AP1, TonEBP, and Sp1: Role of MAPKs

**DOI:** 10.1359/jbmr.091202

**Published:** 2009-07-12

**Authors:** Akihiko Hiyama, Shilpa S Gogate, Sachin Gajghate, Joji Mochida, Irving M Shapiro, Makarand V Risbud

**Affiliations:** 1 Department of Orthopaedic Surgery, Thomas Jefferson University Philadelphia, PA, USA; 2 Department of Orthopaedic Surgery, Surgical Science, Tokai University School of Medicine Bohseidai, Isehara, Kanagawa, Japan

**Keywords:** nucleus pulposus, GlcAT-1, glycosaminoglycan, TGF-β, BMP-2, TonEBP

## Abstract

The goal of the study was to investigate bone morphogenetic protein 2 (BMP-2) and transforming growth factor β (TGF-β) control of the expression of β1,3-glucuronosyl transferase 1 (GlcAT-1), an important regulator of chondroitin sulfate synthesis in cells of the nucleus pulposus. Treatment with both growth factors resulted in induction of GlcAT-1 expression and promoter activity. Deletion analysis indicated that promoter constructs lacking AP1 and TonE sites were unresponsive to growth factor treatment. Experiments using dominant-negative proteins showed that these transcription factors along with Sp1 were required for induction of GlcAT-1 promoter activity. Moreover, when either AP1 or TonE binding sites were mutated, induction was suppressed. Both BMP-2 and TGF-β increased c-Jun and TonEBP expression and phosphorylation of transactivation domains. We investigated the role of the mitogen-activated protein kinase (MAPK) signaling pathway following growth factor treatment; a robust and transient activation of ERK1/2, p38, and JNK was noted. Treatment with MAPK inhibitors blocked BMP-2- and TGF-β-induced AP1 reporter function, GlcAT-1 expression, *and* GAG accumulation. We found that DN-ERK1 but not DN-ERK2 resulted in suppression of growth factor–mediated induction of GlcAT-1 promoter activity; we also showed that p38δ was important in GlcAT-1 activation. Results of these studies demonstrate that BMP-2 and TGF-β regulate GlcAT-1 expression in nucleus pulposus cells through a signaling network comprising MAPK, AP1, Sp1, and TonEBP. It is concluded that by controlling both GAG and aggrecan synthesis, these growth factors positively influence disk cell function. © 2010 American Society for Bone and Mineral Research.

## Introduction

The intervertebral disk is a specialized structure that permits rotation as well as flexure and extension of the human spine. It consists of an outer ligament, the annulus fibrosus, which encloses a gel-like tissue, the nucleus pulposus. While sparse, cells in the nucleus pulposus secrete a complex extracellular matrix that contains fibrillar collagens and the proteoglycan aggrecan. Glycosaminoglycan (GAG) components of the aggrecan molecule provide a robust hydrodynamic system that serves to accommodate applied biomechanical forces.(1,2) Surprisingly, while the importance of aggrecan secretion and function has been discussed by many investigators, mechanisms of control of GAG synthesis are poorly understood.

In the nucleus pulposus, the principal GAG is chondroitin sulfate. Structurally, this molecule is a heteropolysaccharide containing repeating units of *N*-acetyl galactosoamine linked to glucuronic acid. In its fully sulfated form, the molecule exhibits a high charge density, and when hydrated, it assumes a linear configuration. Bound to the aggrecan core protein and associated with hyaluronic acid, the chondroitin sulfate chains form a giant polydispersed supramolecular structure. The high osmotic pressure of the aggregate serves to accommodate applied biomechanical forces.(3)

In earlier studies we and others have shown that nucleus pulposus cells respond to growth factors transforming growth factor β (TGF-β) and bone morphogenetic protein 2 (BMP-2) by upregulating aggrecan and sulfated GAG synthesis.(4,5) However, mechanisms that control chondroitin sulfate chain synthesis in response to these morphogenic factors have not been determined. One possibility is that galactose β1,3-glucuronosyl transferase 1 (GlcAT-1), the enzyme that catalyzes the transfer of glucuronic acid to the core protein Gal–Gal–Xyl–*O*-Ser trisaccharide, a key step in chondroitin sulfate synthesis, is under growth factor control.(6–9) Indeed, our recent finding that GlcAT-1 expression is responsive to tonicity enhancing–binding protein (TonEBP), a transcription factor sensitive to shifts in calcium flux and changes in osmotic pressure, lends support to this hypothesis.(10,11)

The major objective of the investigation was to determine if GlcAT-1 expression is regulated by BMP-2 and TGF-β in nucleus pulposus cells and whether regulation involved TonEBP. We show for the first time that BMP-2 and TGF-β regulate GlcAT-1 expression through activation of multiple transcription factors, including AP1, TonEBP, and Sp1. We also demonstrate that mitogen-activated protein kinase (MAPK) signaling links growth factor activation to these downstream transcription factors. From this perspective, by controlling GAG as well as aggrecan synthesis, BMP-2 and TGF-β promote nucleus pulposus cell function.

## Materials and Methods

### Plasmids and reagents

Human GlcAT-1 reporter plasmids have been reported before.(10) Plasmids were kindly provided by Dr Ben C Ko, University of Hong Kong, People's Republic of China [FLAG-(DN)-TonEBP and FLAG-CMV2(12); DN-TonEBP contains amino acids 157 through 581 of human TonEBP (from clone KIAA0827)], Dr Joan Ferraris, NIH, Bethesda, MD, USA (Gal4-TonEBP-TAD and pFR-Luc),(13) Dr Charles Vinson, NIH (DN-AP1; A-Fos), Dr Silvio Gutkind, NIH (AP1 reporter), Dr Shannon Kenney, University of Wisconsin, Madison, WI, USA (Gal4-c-Jun-TAD), and Dr Gerald Thiel, University of Saarland Medical Center, Germany (DN-Sp1). Plasmid for DN-ERK1 (ERK1K71R) and DN-ERK2 (ERK2K52R) was provided by Dr Melanie Cobb, University of Texas Southwestern Medical Center, Dallas, TX, USA.(14) DN-p38 plasmids (p38αAF, p38β2AF, p38δAF, and p38γAF) were provided by Dr Jiahui Han, Scripps Research Institute, La Jolla, CA, USA. *TonEBP/NFAT5* wild-type and null mouse embryonic fibroblasts (MEFs) (originally from Dr. Steffan N. Ho) were provided by Dr Feng Chen, Washington University, St. Louis, MO, USA. As an internal transfection control, vector pRL-TK (Promega, Madison, WI, USA) containing *Renilla reniformis* luciferase gene was used. The amount of transfected plasmid, the pretransfection period after seeding, and the posttransfection period before harvesting have been optimized for rat nucleus pulposus cells using pSV β-galactosidase plasmid (Promega).(15) Rabbit polyclonal TonEBP antibody was a kind gift from Dr H Moo Kwon, University of Maryland, College Park, MD, USA.

### Isolation of nucleus pulposus cells and treatments of cells

Rat nucleus pulposus cells were isolated using a method reported earlier by Risbud and colleagues.(15) Nucleus pulposus cells were maintained in DMEM and 10% fetal bovine serum (FBS) supplemented with antibiotics. In some experiments, cells were treated with TGF-β3 (10 ng/mL; R&D Systems) or BMP-2 (200 ng/mL, R&D Systems, Minneapolis, MN, USA) with low-molecular-weight heparin (4 µg/mL, Sigma Chemical Company, St. Louis, MO, USA) with or without PD98059 (1 to 30 µM), SKF86002 (1 to 20 µM), and SP60025 (1 to 10 µM) or bisanthracycline (WP631, 50 to 100 nM).

### Real-time PCR analysis

Following treatment, total RNA was extracted from nucleus pulposus cells using RNAeasy minicolumns (Qiagen, Valencia, CA, USA). Before elution from the column, RNA was treated with RNase-free DNAse I. Then 100 ng of total RNA was used as template for real-time polymerase chain reaction (RT-PCR) analysis. Reactions were set up in microcapillary tubes using 1 µL of RNA with 9 µL of a LightCycler FastStart DNA Master SYBR Green I mix (Roche Diagnostics, Indianapolis, IN, USA) to which gene-specific forward and reverse PCR primers were added (GlcAT-1: NCBI no. NM_001128184 forward: 5'-ATGCCCAGTTTGATGCTACTGCAC -3'; reverse: 5'-TGTTCCTCCTGCTTCATCTTCGGT-3'). Each set of samples included a template-free control. PCR reactions were performed in a LightCycler (Roche, Indianapolis, IN, USA) according to the manufacturer's instructions. All the primers used were synthesized by Integrated DNA Technologies, Inc. (Coralville, IA, USA, Littleton, CO, USA).

### Immunofluorescence microscopy

Cells were plated in flat-bottom 96-well plates (4 × 10^3^ cells per well) and treated with BMP-2 and TGF-β for 6 to 24 hours. After incubation, cells were fixed with 4% paraformaldehyde, permeabilized with 0.2% Triton-X 100 in PBS for 10 minutes, blocked with PBS containing 5% FBS, and incubated with antibodies against GlcAT-1 (1:200) (Novus, Littleton, CO, USA) or TonEBP (1:200) at 4°C overnight. As a negative control, cells were reacted with isotype IgG under similar conditions. After washing, the cells were incubated with Alexafluor-488 conjugated anti-mouse secondary antibody (Invitrogen) at a dilution of 1:50 and 10 µM propidium iodide for 1 hour at room temperature. Cells were imaged using a laser scanning confocal microscope (Olympus Fluoview, Tokyo, Japan).

### Nuclear protein extraction and Western blotting

Cells were placed on ice immediately following treatment and washed with ice-cold Hank's balanced salt solution (HBSS). Nuclear proteins were prepared using the CellLytic NuCLEAR extraction kit (Sigma-Aldrich, St. Louis, MO, USA). All the wash buffers and final resuspension buffer included 1 × protease inhibitor cocktail (Pierce, Rockford, IL, USA), NaF (5 mM), and Na_3_VO_4_ (200 µM). Nuclear or total cell proteins were resolved on 8% to 12% SDS-PAGE and transferred by electroblotting to nitrocellulose membranes (Bio-Rad, Hercules, USA). The membranes were blocked with 5% nonfat dry milk in Tris Buffered Saline with Tween 20 (0.1%) (TBST) (50 mM Tris, pH 7.6, 150 mM NaCl, 0.1% Tween 20) and incubated overnight at 4°C in 3% nonfat dry milk in Tris Buffered Saline with Tween 20 (0.1%) (TBST) with the anti-GlcAT-1 antibody (1:500; Novus) or anti-TonEBP antibody (1:3000, from Dr Kwon). Immunolabeling was detected using the enhanced chemiluminescence (ECL) reagent (Amersham Biosciences, Piscataway, NJ, USA).

### Site-directed mutagenesis

GlcAT-1-D reporter plasmid was used to mutate either the TonE site (TTTCCA to TAAAAA) or the AP1 site (CCAGTCACC to AAAAAAACC) or both. Mutants were generated using QuickChange II XL site-directed mutagenesis kit (Stratagene, Wilmington, DE, USA), using forward and reverse primer pairs containing the desired mutation, following the manufacturer's instructions. The mutations were verified by sequencing.

### Transfections and dual luciferase assay

Cells were transferred to 24-well plates at a density of 4 × 10^4^ cells per well 1 day before transfection. To measure the effect of growth factors, cells were transfected with 500 ng of GlcAT-1 reporter plasmids with 500 ng pRL-TK plasmid. To investigate the effect of AP1 or TonEBP or MAPK on GlcAT-1 promoter activity, cells were cotransfected with 100 to 300 ng of A-fos (DN-AP1) or DN-TonEBP (100 to 300 ng) or DN-Sp1 (100 to 300 ng) or DN-MAPK (100 to 300 ng, both ERK and p38) or backbone vector with 400 ng GlcAT-1 reporter and 300 ng pRL-TK plasmid in the presence of TGF-β (10 ng/mL) or BMP-2 (200 ng/mL). For the GAL4 binary assay, cells were cotransfected with 100 ng of pFR-Luc and 100 ng of GAL4dbd or GAL4dbd-TonEBP-TAD (Ton-TAD) or GAL4dbd-cJunTAD (cJun-TAD) and treated with TGF-β (10 ng/mL) or BMP-2 (200 ng/mL). LipofectAMINE 2000 (Invitrogen) was used as a transfection reagent. For each transfection, plasmids were premixed with the transfection reagent. For measuring the effect of MAPK inhibitors on GlcAT-1 reporter activity, 24 hours after transfection, the cells in some wells were treated with inhibitors PD98059 (30 to 50 µM) or SKF86002 (5 to 25 µM) or SP60025 (1 to 10 µM). The next day, the cells were harvested and a Dual-Luciferase reporter assay system (Promega) was used for sequential measurements of firefly and *Renilla* luciferase activities. Quantification of luciferase activities and calculation of relative ratios were carried out using a luminometer (TD-20/20, Turner Designs, Sunnyvale, CA, USA). At least three independent transfections were performed, and all analyses were carried out in triplicate.

### DMMB assay

The proteoglycan content of the cells cultured for 5 days with or without inhibitors was measured as sGAG by colorimetric assay with 1,9-dimethylmethylene blue (DMMB) (Blyscan, Biolcolor, Ltd., Carrickfergus, County Antrim, UK) with chondroitin-4-sulfate as a standard following the manufacturer's instructions. Briefly, GAGs were precipitated from cell extracts and conditioned medium and stained with DMMB, and staining was quantified by measuring absorbance at 656 nm. Results were calculated as GAG (µg)/total DNA (ng) and expressed relative to value obtained for untreated controls.

### Statistical analysis

All measurements were performed in triplicate; data are presented as mean ± SD. Differences between groups were analyzed by Student's *t* test; *p* *<* .05 is considered significant.

## Results

**Fig. 1:**
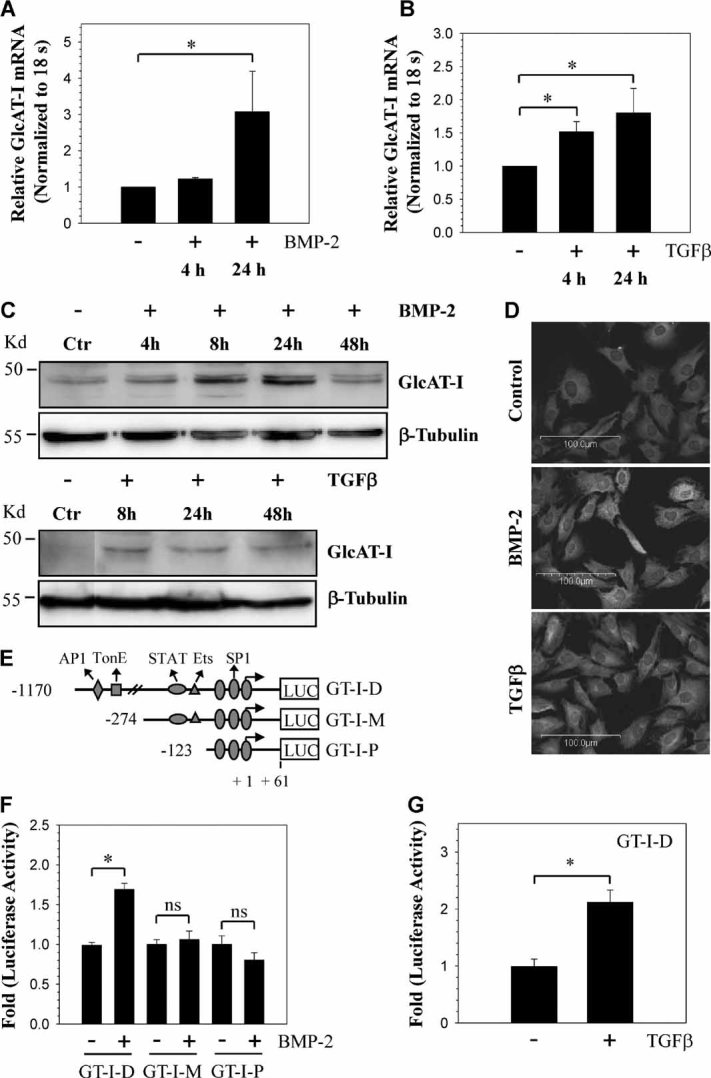
RT-PCR analysis of *GlcAT-1* expression by cells treated with (*A*) BMP-2 and (*B*) TGF-β for 4 to 24 hours. There was a time-dependent change in mRNA expression following treatment. At 4 hours, *GlcAT-1* expression was increased only in TGF-β-treated cells; at 24 hours, there was increased expression in both treatment groups. (*C*) Western blot analysis of GlcAT-1 expression by nucleus pulposus cells following treatment with BMP-2 and TGF-β for 4 to 48 hours. Note the increased GlcAT-1 expression 8 hours after treatment. (*D*) Immunofluorescent analysis of nucleus pulposus cells treated with BMP-2 and TGF-β. Cells showed increased GlcAT-1 expression 24 hours after the treatment. (*E*) Cartoon showing map of successive PCR-generated 5' deletion constructs of the human GlcAT-1 promoter; the transcription start site is marked as +1. The GT-1-D construct consists of a 1231-bp fragment (−1170/+61), whereas GT-1-M and GT-1-P constructs contain a 335-bp fragment (−274/+61) and a 184-bp fragment (−123/+ 61), respectively. The tonE site is shown as a square, the Sp1 sites are indicted as ovals, and the AP1-binding motif is shown as a diamond. (*F*, *G*) GlcAT-1 reporter activities measured following (*F*) BMP-2 and (*G*) TGF-β treatment. Treatment resulted in induction in GT-1-D but not GT-1-M or GT-1-P reporter activity. Values shown are mean ± SD, *n* = 3. *p < .05.

To explore the premise that BMP-2 and TGF-β regulated *GlcAT-1* expression, nucleus pulposus cells were treated with each of the factors and expression of *GlcAT-1* was analyzed. Figure 1*A, B* shows that treatment with both BMP-2 and TGF-β for 24 hours resulted in increased *GlcAT-1* mRNA levels in nucleus pulposus cells. However, simultaneous treatment of nucleus pulposus cells with TGF-β and BMP-2 did not synergize *GlcAT-1* mRNA expression (Supplemental Fig. 1). We also measured GlcAT-1 protein expression using Western blot analysis. Figure 1*C* shows that there was increased expression of GlcAT-1 protein as early as 8 hours after the treatment with both BMP-2 and TGF-β; expression level remained elevated till 24 hours but returned to the basal state 48 hours after the treatment. In addition, we studied expression of GlcAT-1 in nucleus pulposus cells using immunofluorescence microscopy. Growth factor treatment resulted in increased GlcAT-1 protein expression (Fig. 1*D*). In all cases, staining was localized to the cytosol (Fig. 1*D*). In contrast to the nucleus pulposus, treatment of annulus fibrosus cells with either of the growth factors did not change *GlcAT-1* transcription and mRNA levels (Supplemental Fig. 1*B*).

To investigate the regulation of expression, we generated luciferase reporter constructs containing −1170/+61 bp (pGT-I-D), −274/+61 bp (pGT-I-M), and −123/+61 bp (pGT-I-P) fragments of the human GlcAT-1 promoter (Fig. 1*E*) and measured their activities. When cells were treated with BMP-2, there was an increase in activity of −1170/+61 bp GlcAT-1 promoter (pGT-1-P); the shortest promoter fragments (pGT-1-M and pGT-1-P), lacking TonE and AP1 sites, did not show any change in activity (Fig. 1*F*). Similarly, only the pGT-1-D reporter responded to TGF-β treatment (Fig. 1*G*).

**Fig. 2:**
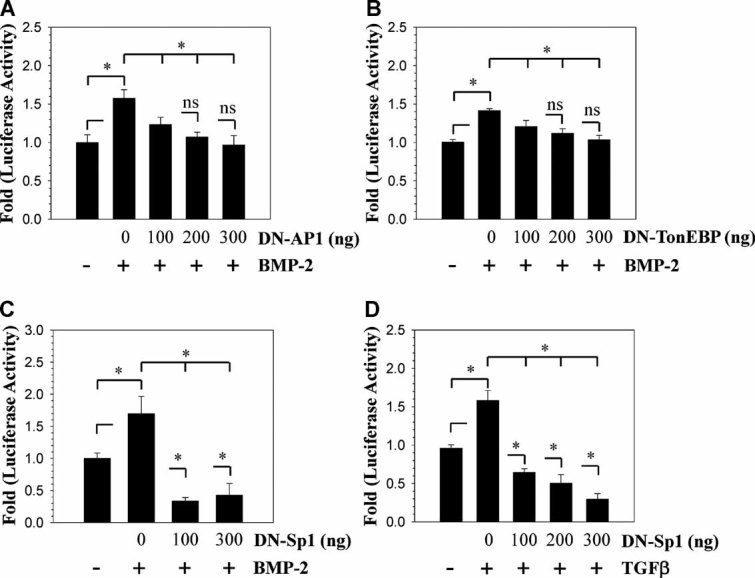
BMP-2 and TGF-β regulate GlcAT-1 promoter activity through AP1, TonEBP, and Sp1. (*A–C*) Effect of BMP-2 treatment on cells transfected with GlcAT-1-D reporter construct along with (*A*) DN-AP1, (*B*) DN-TonEBP, and (*C*) DN-Sp1 or respective empty backbone vectors. Note that the expression of DN proteins resulted in a complete suppression of growth factor–mediated induction in GlcAT-1 promoter activity. When Sp1 function was blocked, GlcAT-1-D activity was suppressed below basal levels. (*D*) Cotransfection with DN-Sp1 significantly suppresses TGF-β-mediated induction in GlcAT-1-D reporter activity. Values shown are mean ± SD, *n* = 3. *p < .05.

**Fig. 3:**
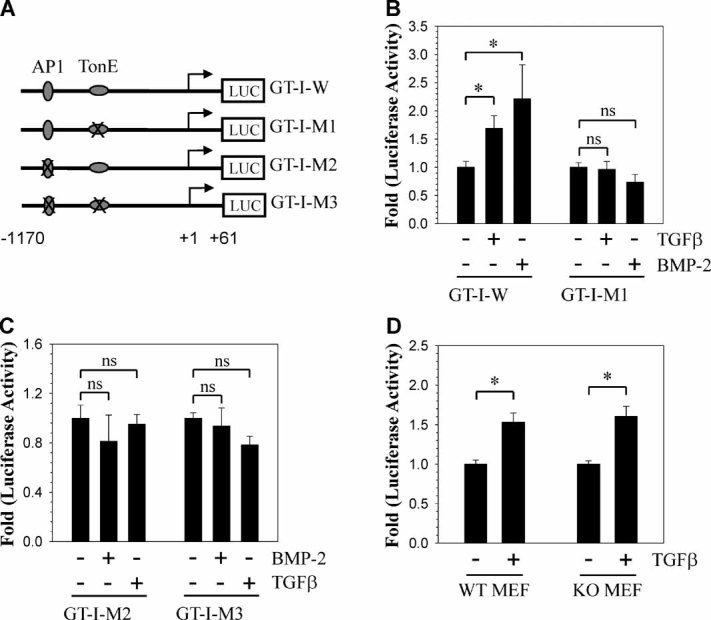
BMP-2 and TGF-β regulate GlcAT-1 promoter activity through AP1 and TonEBP in a cell type–specific manner. (*A*) Reporters used to study effect of individual and combined mutation in TonE and AP1 motifs on GlcAT-1 promoter activity. (*B*, *C*) Nucleus pulposus cells were transfected with wild-type GT-1-D (GT-1-W) or mutant GT-1-D (GT-1-M) reporter plasmids, and the induction of luciferase activity was determined following BMP-2 and TGF-β treatment. Treatment caused an induction of wild-type reporter activity (*B*), whereas the mutant reporters failed to increase their activities. (*D*) Measurement of GlcAT-1-D reporter activity in TonEBP wild-type (WT) and null (KO) MEFs after TGF-β treatment. Both WT and KO cells showed induction in reporter activity. Values shown are mean ± SD, *n* = 3. *p < .05.

To investigate if AP1 and TonEBP participated in BMP-2- and TGF-β-mediated induction of GlcAT-1 promoter activity, nucleus pulposus cells were transiently cotransfected with plasmids encoding DN-TonEBP or DN-AP1 (A-fos). Figure 2*A* shows that forced expression of DN-AP1 completely abolished BMP-2 induction of GlcAT-1 promoter activity. A significant inhibitory effect of DN-AP1 expression on GlcAT-1 reporter activity was seen at a dose of 100 ng (Fig. 2*A*). Similarly, coexpression of DN-TonEBP1 (Fig. 2*B*) and DN-Sp1 (Fig. 2*C*) resulted in a decrease in GlcAT-1-D promoter activity in the presence of BMP-2. Similar to BMP-2, TGF-β-mediated induction in promoter activity was suppressed by DN-Sp1 (Fig. 2*D*) and DN-TonEBP (not shown). We then evaluated whether BMP-2- and TGF-β-mediated induction in GlcAT-1 promoter activity was dependent on interactions with AP1 and TonEBP motifs in nucleus pulposus cells. For this purpose, we generated a series of constructs that contained a mutation in either TonE or AP1 or both of these motifs (Fig. 3*A*). Following treatment, nucleus pulposus cells transfected with wild-type reporter plasmid evidenced induction of activity (Fig. 3*B*). In contrast, when a plasmid harboring either a single or a double mutation was used, induction of the reporter was completely blocked (Fig. 3*B, C*). We further used TonEBP wild-type and null MEFs to investigate whether TonEBP was required for growth factor–mediated induction in GlcAT-1 promoter activity. In both wild-type and null MEFs, TGF-β induced GlcAT-1 promoter activity (Fig. 3*D*).

**Fig. 4:**
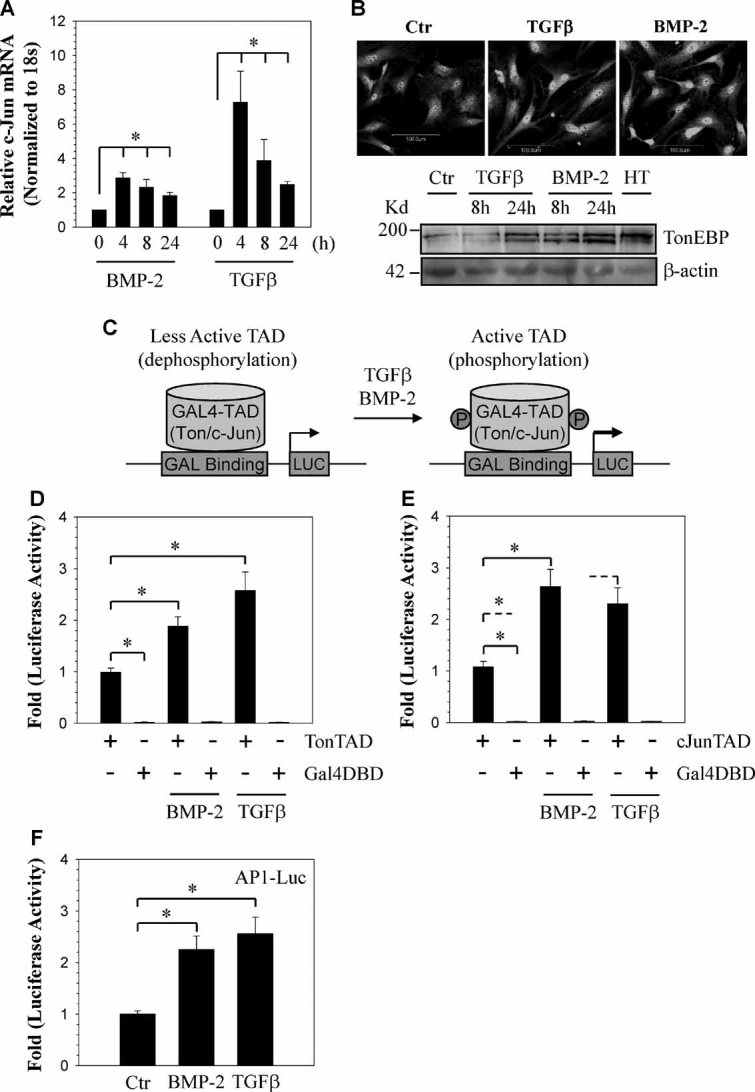
Effect of BMP-2 and TGF-β on AP1 and TonEBP expression and activity. Nucleus pulposus cells were treated with the growth factors and c-*Jun* and *TonEBP* expression was measured. (*A*) Treatment resulted in significant increase in c-*Jun* mRNA expression. (*B*) Immunofluorescence and Western blot analysis of treated cells. Note the increase in TonEBP protein after both BMP-2 and TGF-β treatment. As expected, cells cultured under hyperosmotic conditions (HT: 450 mOsmol/kg) showed a high induction in TonEBP expression. Ctr = control. (*C*) BMP-2 and TGF-β regulates transactivation activity of TonEBP and AP1 (TAD phosphorylation) in nucleus pulposus cells. Cells were transfected with GAL4 binary reporter system comprising either (*D*) GAL4Ton-TAD and (*E*) Gal4cJun-TAD or the empty GAL4DBD vector with pFR-Luc vector and treated with the growth factors. Treatment significantly increased both TonEBP-TAD and cJun-TAD activity. In all instances, when transfected with the empty GAL4DBD vector, luciferase activity was extremely low, and there was no change. (*F*) Effect of growth factor treatment on regulation of AP1. Both BMP-2 and TGF-β resulted in upregulation of AP1-responsive reporter function, indicating activation of AP1 signaling. Data represents mean ± SD of three independent experiments performed in triplicate. *p < .05.

We then determined if BMP-2 and TGF-β modulated AP1 and TonEBP expression and activity in nucleus pulposus cells. Figure 4*A* shows that growth factor treatment resulted in a significant increase in c-*Jun* mRNA expression. Likewise, both immunofluorescence and Western blot analysis indicated that there was a concomitant increase in TonEBP expression: The increase in protein level was evident as early as 8 hours, and it remained high at 24 hours (Fig. 4*B*). Next, studies were preformed to determine if growth factor treatment modulated the phosphorylation status of TonEBP-TAD and cJun-TAD in nucleus pulposus cells (Fig. 4*C*). We found that when cells were treated with either BMP-2 or TGF-β, there was significant activation of both TonTAD (Fig. 4*D*) and cJun-TAD (Fig. 4*E*) activity. Cells that were transfected with empty Gal4dbd exhibited minimal luciferase activity, which did not change after the growth factor treatment (Fig. 4*D, E*). In addition, we measured AP1-responsive reporter activity in nucleus pulposus cells. Figure 4*F* shows that treatment with either BMP-2 or TGF-β resulted in significant induction in AP1 reporter activity.

**Fig. 5:**
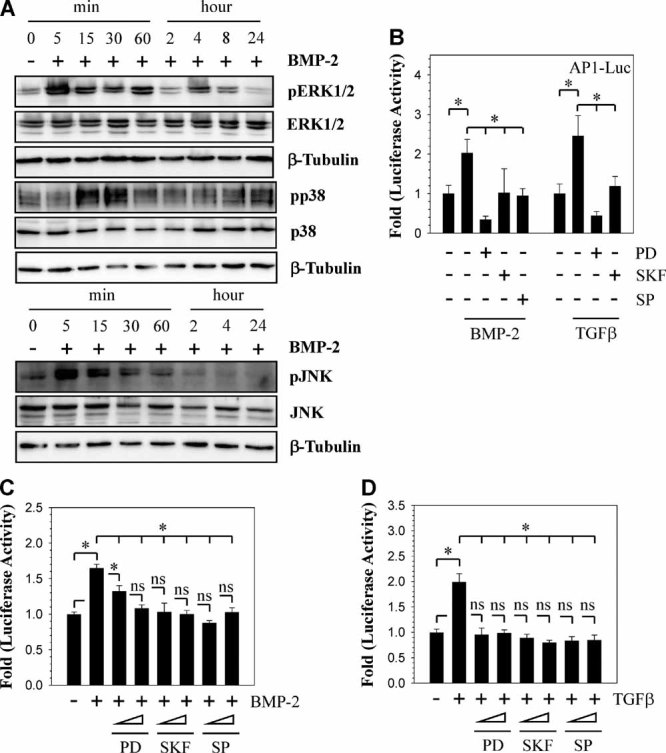
MAPK regulation of GlcAT-1 expression. (*A*) Western blot analysis of ERK, p38, and JNK activation following treatment of nucleus pulposus cells with BMP-2. Growth factor treatment induced phosphorylation of ERK1/2, p38, and JNK within 15 minutes, and levels returned to baseline after 24 hours. Nucleus pulposus cells were transfected with (*B*) AP1 and (*C*, *D*) GlcAT-1 reporter plasmid. Transfected cells were treated with BMP-2 and TGF-β with or without MAPK inhibitors PD98059 (PD; 30 to 50 µM), SKF86002 (SKF; 5 to 20; µM), and SP600125 (SP; 1 to 10; µM) for 24 hours, and luciferase activity was measured. Treatment with inhibitors caused complete suppression of AP1 and GlcAT-1 reporter activity. Values shown are of three independent experiments; mean ± SD. *p < .05.

**Fig. 6:**
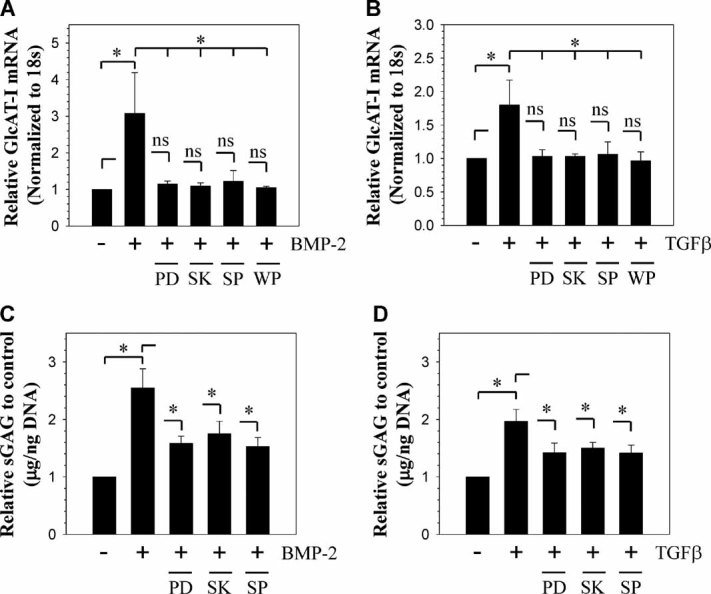
MAPK regulates GlcAT-1 expression and GAG synthesis. (*A*, *B*) RT-PCR analysis of *GlcAT-1* expression by cells treated with BMP-2 (*A*) or TGF-β (*B*) with or without MAPK (PD, SKF, and SP) or SP1 (WP631) inhibitors for 4 to 24 hours. There was a suppression of *GlcAT-1* mRNA expression following inhibitor treatment. In the absence of inhibitors, both the growth factors increased expression of the gene. (*C, D*) GAG accumulation by nucleus pulposus cells treated with BMP-2 (*C*) or TGF-β (*D*) with or without MAPK (PD, SKF, and SP) inhibitors for 5 days. Both the growth factors increase GAG accumulation by nucleus pulposus cells; there is a partial decrease in GAG content when inhibitors are used. Values shown are of three independent experiments; mean ± SD. *p < .05.

We further investigated the role of upstream signaling pathways that controlled TonEBP and AP1 activation by BMP-2 and TGF-β in nucleus pulposus cells. Since MAPKs are one of the key downstream effectors of TGF-β and BMP-2, we evaluated the role of this signaling pathway in the activation of GlcAT-1 promoter activity in nucleus pulposus cells. Figure 5*A* shows that following BMP-2 treatment, there was a rapid increase in pERK1/2, p38, and JNK levels. Activation was maximum between 5 and 30 minutes and declined rapidly thereafter, returning to basal levels by 24 hours. To confirm that growth factor–dependent transient activation of MAPK signaling was required for AP1 activation, we measured BMP-2- and TGF-β-induced AP1 reporter activity in the presence of MAPK inhibitors. Figure 5*B* shows that growth factor treatment significantly increased the activity of AP1 reporter; this activity was completely suppressed in the presence of MAPK inhibitors PD98059, SKF86002, and SP600125. To further validate the role of MAPK signaling in controlling growth factor–mediated GlcAT-1 expression, we measured its promoter activity and mRNA expression levels in the presence of inhibitors. Treatment with MAPK inhibitors in the presence of BMP-2 and TGF-β resulted in suppression of induction in GlcAT-1 promoter activity (Fig. 5*C, D*) and *GlcAT-1* mRNA expression (Fig. 6*A, B*). In addition, the Sp1 inhibitor WP631 also suppressed growth factor–mediated *GlcAT-1* mRNA expression (Fig. 6*A, B*). MAPK inhibition also resulted in a decrease in accumulation of sGAG stimulated by both BMP-2 (Fig. 6*C*) and TGF-β (Fig. 6*D*).

**Fig. 7:**
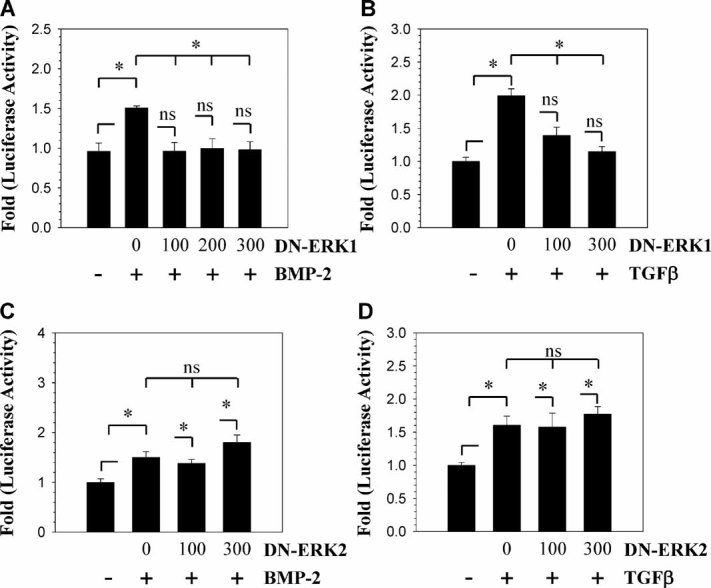
MAPK regulates growth factor–induced GlcAT-1 promoter activity. Nucleus pulposus cells were cotransfected with GlcAT-1 (GT-1-D) reporter plasmid along with either (*A*, *B*) DN-ERK1 or (*C*, *D*) DN-ERK2 or respective empty backbone vectors (pSG5 or pcDNA3). Transfected cells were treated with either BMP-2 (*A*, *C*) or TGF-β (B, *D*), and reporter activity was measured 24 hours after the treatment. When DN-ERK1 was cotransfected with the GlcAT-1 reporter, the growth factor–mediated induction of the reporter was significantly suppressed. On the other hand, cotransfection with DN-ERK2 did not result in suppression of the growth-mediated increase in promoter activity. Values shown are of three independent experiments; mean ± SD. *p < .05.

**Fig. 8:**
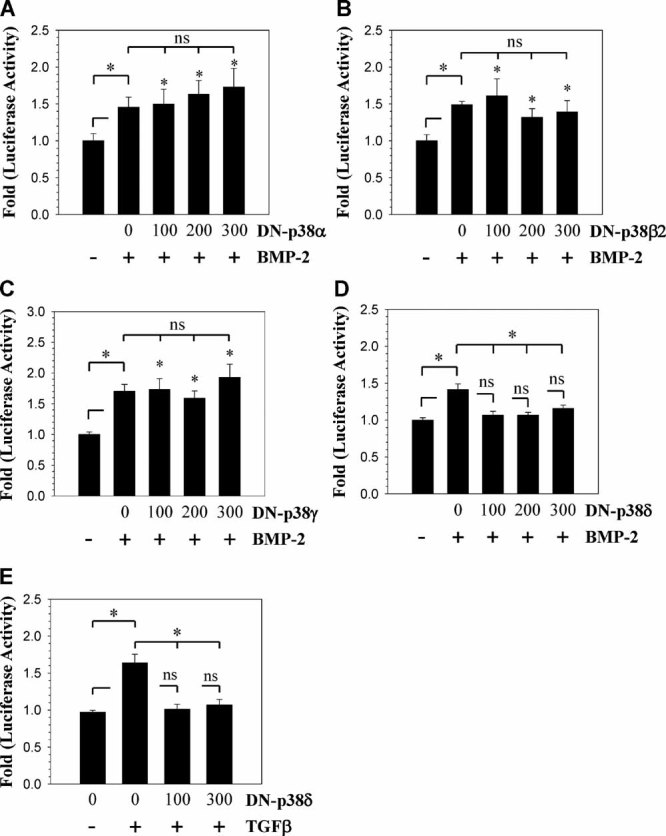
MAPK regulation of growth factor–mediated GlcAT-1 promoter activity. Nucleus pulposus cells were cotransfected with GlcAT-1 (GT-1-D) reporter plasmid along with either (*A*) p38α, (*B*) p38β2, (*C*) p38γ, (*D*, *E*) p38δ or empty backbone vector pcDNA3. Transfected cells were treated with either BMP-2 or TGF-β, and reporter activity was measured 24 hours after the treatment. Cotransfection of DN-p38δ with the GlcAT-1 reporter resulted in suppression of growth factor–mediated induction of the reporter activity. On the other hand, cotransfection with p38α, p38β2, or p38γ did not result in suppression of growth factor–mediated increase in the promoter activity. Values shown are of three independent experiments; mean ± SD. *p < .05.

Finally, we used the loss-of-function approach to ascertain which isoforms of ERK and p38 are required for maintenance of GlcAT-1 promoter activity in nucleus pulposus cells. When nucleus pulposus cells were cotransfected with the DN-ERK1 expression plasmid in the presence of both BMP-2 (Fig. 7*A*) and TGF-β (Fig. 7*B*), there was a dose-dependent suppression in GlcAT-1 promoter activity. Inhibition of reporter activity was evident even when the DN-ERK1 plasmid concentration was reduced to 100 ng. On the other hand, no change in promoter activity was observed when DN-ERK2 was used (Fig. 7*C, D*). Similarly, cotransfection with p38δ (Fig. 8*D*) but not p38α (Fig. 8*A*), p38β2 (Fig. 8*B*), or p38γ (Fig. 8*C*) resulted in suppression of reporter activity in the presence of BMP-2. Likewise, TGF-β-mediated induction in GlcAT-1 promoter activity was suppressed by with DN-p38δ (Fig. 8*E*).

## Discussion

The experiments described in this investigation demonstrated for the first time that expression of GlcAT-1, a key enzyme required for chondroitin sulfate biosynthesis, was regulated by BMP-2 and TGF-β. When treated with growth factors, there was induction of GlcAT-1 promoter activity and expression. Our studies revealed that regulation was mediated through AP1, Sp1, and TonEBP (NFAT5), a transcription factor that serves to promote the expression of GlcAT-1 and enhance the expression of aggrecan.(10,11) A second major observation was that BMP-2 and TGF-β both signal through specific MAPKs, thereby promoting AP1, Sp1, and TonEBP transcriptional activity. Thus, from a functional perspective, signaling is mediated by members of the TGF-β family of proteins as well as environmental stimuli that include osmotic pressure and biomechanical loading (calcium flux). These signals are transduced by TonEBP, which, when activated, enhances the synthesis of hydrated proteoglycans by cells of the intervertebral disk. In this way, by controlling both GAG and aggrecan synthesis, cells of the nucleus pulposus can autoregulate their own osmotic environment and accommodate mechanical loading.

TGF-β and BMP-2 have been shown to regulate proteoglycan synthesis by cells of the intervertebral disk.(4,5,16) In addition, it is known that both these growth factors stimulate chondroitin sulfate biosynthesis.(4,5,16) However, the mechanism of regulation of GAG expression is not understood. We focused on the role of GlcAT-1 in this process because this enzyme catalyzes a critical step in the synthesis of chondroitin sulfate: the transfer of glucuronic acid to the core protein Gal–Gal–Xyl–*O*-Ser trisaccharide.(6,8) To ascertain whether TGF-β and BMP-2 regulated the expression of GlcAT-1 in nucleus pulposus cells, we measured both transcript and protein expression. Following treatment with each of these growth factors, there was robust expression of GlcAT-1 at both the mRNA and protein levels. This finding indicated that regulation was at the transcriptional level. We performed deletion analysis to determine the importance of both the TonEBP and AP1 sites.(10) Since these studies indicated that there was loss of promoter activity, it was concluded that both TonEBP and AP1 were required for GlcAT-1 expression in nucleus pulposus cells. Loss-of-function experiments using dominant-negative constructs confirmed that GlcAT-1 activation was regulated by AP1 and TonEBP as well as by Sp1. Not surprisingly, compared with AP1 and TonEBP, the effect of Sp1 inhibition on GlcAT-1 reporter activity was profound. This is probably linked to the need for Sp1 to maintain basal transcription activity in nucleus pulposus cells.(10) When GlcAT-1 reporters that contained a mutant TonE and/or AP1 were treated with BMP-2 and TGF-β, there was failure to increase activity. This observation lent further strength to the findings reported earlier and indicated that AP1 and TonEBP in *conjunction with Sp1* regulated GlcAT-1 expression.

Since the nucleus pulposus is unique both embryologically and functionally, it was important to ascertain if regulatory control of GlcAT-1 was specific for this cell type.(17) For this purpose, we used MEFs derived from *TonEBP* null mice and annulus fibrosus cells. Not surprisingly, the null MEFs displayed decreased basal GlcAT-1 promoter activity; however, when treated with growth factors, an increase in GlcAT-1 reporter activity was observed. In contrast, annulus fibrosus cells were unresponsive and did not induce GlcAT-1 expression. This result pointed to distinct differences in responses of MEFs, annulus fibrosus, and nucleus pulposus cells to growth factor treatment. It is not unreasonable to assume that TonEBP contributes in GlcAT-1 regulation in a context- and cell type–specific manner. Our result is also in agreement with a recent study in which it was shown that there was no effect of BMP-2 treatment on GAG synthesis by annulus fibrosus cells.(18) We conclude that TGF-β and BMP-2 promote recruitment of TonEBP to the GlcAT-1 promoter and that it may involve regulatory mechanisms such as cofactors unique to cells of the nucleus pulposus. Furthermore, the results of these functional studies indicate that in addition to regulating levels of osmolytes and the water transporter aquaporin2, by controlling the expression of GlcAT-1, TonEBP adapts nucleus pulposus cells to the mechanically and osmotically challenging conditions of the disk.(10,11,19,20)

The mechanism of activation of TonEBP is not completely understood.(21–23) Recent studies indicate that in nucleus pulposus cells, aside from osmolarity, calcium regulates TonEBP activity and expression in a unique fashion.(10,11,20) Indeed, one effect of TGF-β is the initiation of calcium transients.(24) Whether these transients play a role in activating TonEBP has not been determined. Nevertheless, it is clear that irrespective of changes in calcium flux, TGF-β and BMP-2 serve to upregulate TonEBP expression as well as its transcriptional activity possibly in a cell/tissue-specific manner. However, these studies did not delineate the underlying signaling mechanisms responsible for the increase in TonEBP expression and activity. It is likely that signaling is mediated via the AP1 complex. Thus, following stimulation with BMP-2 and TGF-β, there was a concomitant increase in expression and activity of c-Jun, an important constituent of the AP1 signaling complex.(25,26) That there was robust activation of AP1 was evident from studies of the activity of the AP1-responsive reporter. We noted that following the growth factor treatment, the nucleus pulposus cells induced AP1 reporter activity.

To delineate how these growth factors transduce AP1, TonEBP, and Sp1 in nucleus pulposus cells, we focused first on the MAPK pathways. In earlier studies we noted the importance of this pathway in regulating the functional and survival activities of cells of both the nucleus pulposus and annulus fibrosus.(4,27) Indeed, Tsai and colleagues have shown that both ERK and p38 signaling were required for hypertonic activation of TonEBP.(20) Pertaining to the current investigation, MAPK activation of AP1 has been documented in other cell types.(28,29) Here we used a loss-of-function approach to evaluate the relationship between the MAPK signaling pathway and AP1 activation in nucleus pulposus cells. Suppression of ERK, p38, and JNK signaling activity by pharmacologic inhibitors resulted in a complete loss of growth factor–induced AP1 reporter activity; importantly, we observed when MAPK and Sp1 inhibitors were used, there was a decrease in GlcAT-1 promoter activity and expression as well GAG accumulation. While we did not study the mechanism of Sp1 activation, Barré and colleagues reported that calcium ions promoted Sp1 activation through MEK/ERK signaling and its subsequent binding to a conserved motif in the GlcAT-1 promoter.(30) Noteworthy, in an earlier study we confirmed that Sp1 activity was required for calcium-mediated induction of GlcAT-1 activity in nucleus pulposus cells.(10) However, we noted that neither growth factor influenced the activity of GlcAT-1-P construct that lacked the AP1 and TonE motifs. Since this construct contained Sp1 motifs, it is clear that Sp1 activation alone is insufficient for induction of GlcAT-1 promoter activity in nucleus pulposus cells.(10,23) Thus it is likely that BMP-2 and TGF-β enhance GlcAT-1 promoter activity through the MAPK signaling pathway and require AP1, TonEBP, and Sp1.

We determined which isoforms of ERK and p38 were important for growth factor–mediated induction of GlcAT-1. Forced expression of DN-ERK1 and DN-p38δ resulted in a significant inhibition of GlcAT-1 reporter activity. Interestingly, the p38α isoform that promotes tonicity-dependent activation of TonEBP was ineffective.(20) In contrast, the p38δ isoform that has been shown to suppress hypertonic activation of TonEBP(31) modulated GlcAT-1 promoter activity. Together these observations provide two new insights concerning nucleus pulposus cell function. First, TonEBP activity depends on a complex signaling network. Second, loss-of-function techniques used in the investigation confirmed the importance of ERK, p38, and JNK in regulating the transcriptional response of nucleus pulposus cells to growth factor treatment.

In summary, we have shown that BMP-2 and TGF-β regulate expression of GlcAT-1, a critical enzyme required for GAG synthesis through a transcriptional network comprising AP1, TonEBP, Sp1, and MAPK signaling pathways. In addition, these studies show that TonEBP expression is regulated by growth factors that are known to regulate nucleus pulposus cell function. Our ongoing studies are aimed at testing a hypothesis that changes in the activity of GlcAT-1 are linked to the development of degenerative disk disease.

## References

[1] Feng H, Danfelter M, Strömqvist B, Heinegård D (2006). Extracellular matrix in disc degeneration. J Bone Joint Surg Am..

[2] Setton LA, Chen J (2006). Mechanobiology of the intervertebral disc and relevance to disc degeneration. J Bone Joint Surg Am..

[3] Ng L, Grodzinsky AJ, Patwari P, Sandy J, Plaas A, Ortiz C (2003). Individual cartilage aggrecan macromolecules and their constituent glycosaminoglycans visualized via atomic force microscopy. J Struct Biol..

[4] Risbud MV, Di Martino A, Guttapalli A (2006). Toward an optimum system for intervertebral disc organ culture: TGF-β3 enhances nucleus pulposus and annulus fibrosus survival and function through modulation of TGF-β-R expression and ERK signaling. Spine..

[5] Li J, Yoon ST, Hutton WC (2004). Effect of bone morphogenetic protein-2 (BMP-2) on matrix production, other BMPs, and BMP receptors in rat intervertebral disc cells. J Spinal Disord Tech..

[6] Kitagawa H, Ujikawa M, Sugahara K (1996). Developmental changes in serum UDP-GlcA:chondroitin glucuronyltransferase activity. J Biol Chem..

[7] Venkatesan N, Barré L, Benani A (2004). Stimulation of proteoglycan synthesis by glucuronosyltransferase-I gene delivery: a strategy to promote cartilage repair. Proc Natl Acad Sci U S A..

[8] Bai X, Wei G, Sinha A, Esko JD (1999). Chinese hamster ovary cell mutants defective in glycosaminoglycan assembly and glucuronosyltransferase I. J Biol Chem..

[9] Gouze JN, Bordji K, Gulberti S (2001). Interleukin-1β down-regulates the expression of glucuronosyltransferase I, a key enzyme priming glycosaminoglycan biosynthesis: influence of glucosamine on interleukin-1β-mediated effects in rat chondrocytes. Arthritis Rheum..

[10] Hiyama A, Gajghate S, Sakai D, Mochida J, Shapiro IM, Risbud MV (2009). Activation of TonEBP by calcium controls β,3-glucuronosyltransferase I expression, a key regulator of glycosaminoglycan synthesis in cells of the intervertebral disc. J Biol Chem..

[11] Tsai TT, Danielson KG, Guttapalli A (2006). TonEBP/OREBP is a regulator of nucleus pulposus cell function and survival in the intervertebral disc. J Biol Chem..

[12] Lam AK, Ko BC, Tam S (2004). Osmotic response element–binding protein (OREBP) is an essential regulator of the urine concentrating mechanism. J Biol Chem..

[13] Ferraris JD, Williams CK, Persaud P, Zhang Z, Chen Y, Burg MB (2002). Activity of the TonEBP/OREBP transactivation domain varies directly with extracellular NaCl concentration. Proc Natl Acad Sci U S A..

[14] Robinson MJ, Harkins PC, Zhang J (1996). Mutation of position 52 in *ERK2* creates a nonproductive binding mode for adenosine 5'-triphosphate. Biochemistry..

[15] Risbud MV, Guttapalli A, Stokes DG (2006). Nucleus pulposus cells express HIF-1α under normoxic culture conditions: a metabolic adaptation to the intervertebral disc microenvironment. J Cell Biochem..

[16] Walsh AJ, Bradford DS, Lotz JC (2004). In vivo growth factor treatment of degenerated intervertebral discs. Spine..

[17] Choi KS, Cohn MJ, Harfe BD (2008). Identification of nucleus pulposus precursor cells and notochordal remnants in the mouse: implications for disk degeneration and chordoma formation. Dev Dyn..

[18] Gilbertson L, Ahn SH, Teng PN, Studer RK, Niyibizi C, Kang JD (2008). The effects of recombinant human bone morphogenetic protein-2, recombinant human bone morphogenetic protein-12, and adenoviral bone morphogenetic protein-12 on matrix synthesis in human annulus fibrosis and nucleus pulposus cells. Spine J..

[19] Gajghate S, Hiyama A, Shah M (2009). Osmolarity and intracellular calcium regulate aquaporin2 expression through TonEBP in nucleus pulposus cells of the intervertebral disc. J Bone Miner Res..

[20] Tsai TT, Guttapalli A, Agrawal A, Albert TJ, Shapiro IM, Risbud MV (2007). MEK/ERK signaling controls osmoregulation of nucleus pulposus cells of the intervertebral disc by transactivation of TonEBP/OREBP. J Bone Miner Res..

[21] Jeon US, Kim JA, Sheen MR, Kwon HM (2006). How tonicity regulates genes: story of TonEBP transcriptional activator. Acta Physiol (Oxf)..

[22] Trama J, Lu Q, Hawley RG, Ho SN (2000). The NFAT-related protein NFATL1 (TonEBP/NFAT5) is induced upon T cell activation in a calcineurin-dependent manner. J Immunol..

[23] Li SZ, McDill BW, Kovach PA (2007). Calcineurin-NFATc signaling pathway regulates AQP2 expression in response to calcium signals and osmotic stress. Am J Physiol Cell Physiol..

[24] Nesti LJ, Caterson EJ, Li WJ (2007). TGF-beta1 calcium signaling in osteoblasts. J Cell Biochem..

[25] Hall MC, Young DA, Waters JG (2003). The comparative role of activator protein 1 and Smad factors in the regulation of *Timp-1* and *MMP-1* gene expression by transforming growth factor-β1. J Biol Chem..

[26] Zhao L, Yang S, Zhou GQ (2006). Downregulation of cAMP-dependent protein kinase inhibitor γ is required for BMP-2-induced osteoblastic differentiation. Int J Biochem Cell Biol..

[27] Risbud MV, Fertala J, Vresilovic EJ, Albert TJ, Shapiro IM (2005). Nucleus pulposus cells upregulate PI3K/Akt and MEK/ERK signaling pathways under hypoxic conditions and resist apoptosis induced by serum withdrawal. Spine..

[28] Aggeli IK, Gaitanaki C, Beis I (2006). Involvement of JNKs and p38-MAPK/MSK1 pathways in H_2_O_2_-induced upregulation of heme oxygenase-1 mRNA in H9c2 cells. Cell Signal..

[29] Wang HH, Hsieh HL, Wu CY, Sun CC, Yang CM (2009). Oxidized low-density lipoprotein induces matrix metalloproteinase-9 expression via a p42/p44 and JNK-dependent AP-1 pathway in brain astrocytes. Glia..

[30] Barré L, Venkatesan N, Magdalou J, Netter P, Fournel-Gigleux S, Ouzzine M (2006). Evidence of calcium-dependent pathway in the regulation of human β1,3-glucuronosyltransferase-1 (*GlcAT-1*) gene expression: a key enzyme in proteoglycan synthesis. FASEB J..

[31] Zhou X, Ferraris JD, Dmitrieva NI, Liu Y, Burg MB (2008). MKP-1 inhibits high NaCl-induced activation of p38 but does not inhibit the activation of TonEBP/OREBP: opposite roles of p38α and p38δ. Proc Natl Acad Sci USA..

