# Supplementary material for: BMP-2 and TGF-β Stimulate Expression of β1,3-Glucuronosyl Transferase 1 (GlcAT-1) in Nucleus Pulposus Cells Through AP1, TonEBP, and Sp1: Role of MAPKs
Source: J Bone Miner Res. 2009 Jul 12;25(5):1179–90. doi: 10.1359/jbmr.091202 (PMC3153993; doi:10.1359/jbmr.091202)
Supplement: Supplementary file 1 [file jbmr0025-1179-SD1.ppt]

## Slide 1
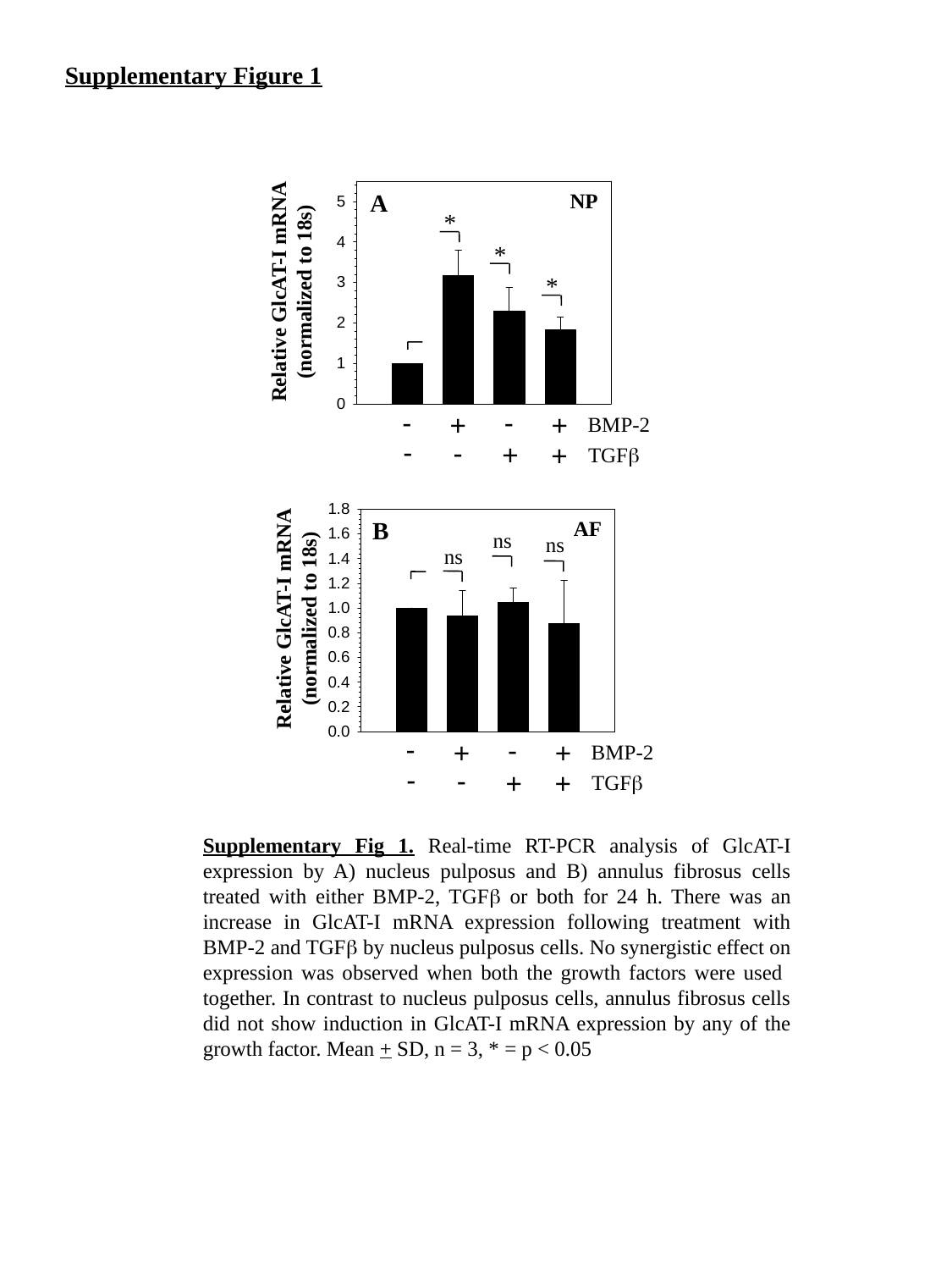

Supplementary Figure 1
NP
A
*
*
Relative GlcAT-I mRNA
(normalized to 18s)
*
-
-
+
+
BMP-2
-
-
+
+
TGFβ
B
AF
ns
ns
ns
Relative GlcAT-I mRNA
(normalized to 18s)
-
-
+
+
BMP-2
-
-
+
+
TGFβ
Supplementary Fig 1. Real-time RT-PCR analysis of GlcAT-I expression by A) nucleus pulposus and B) annulus fibrosus cells treated with either BMP-2, TGFβ or both for 24 h. There was an increase in GlcAT-I mRNA expression following treatment with BMP-2 and TGFβ by nucleus pulposus cells. No synergistic effect on expression was observed when both the growth factors were used together. In contrast to nucleus pulposus cells, annulus fibrosus cells did not show induction in GlcAT-I mRNA expression by any of the growth factor. Mean + SD, n = 3, * = p < 0.05
